# Outcome of a conservatively treated large Pipkin I fracture dislocation: a case report

**DOI:** 10.1093/jscr/rjad513

**Published:** 2023-09-18

**Authors:** Omar Alfreihi, Bander S Alrashedan, Hamid T Aljohani, Sarah O Alturaisi, Jameel Mahmoud, Hani S Serhan

**Affiliations:** Department of Orthopedic Surgery, Prince Sultan Military Medical City, Al MPR7+4CQ, Makkah, Mukarramah Rd, As Sulimaniyah, 12233, Riyadh, Saudi Arabia; Department of Orthopedic Surgery, Ministry of Health (MOH), King Saud Medical City, Ulaishah, Al Imam Abdul Aziz Ibn Muhammad Ibn Saud, 12746, Riyadh, Saudi Arabia; Department of Orthopedic Surgery, Prince Sultan Military Medical City, Al MPR7+4CQ, Makkah, Mukarramah Rd, As Sulimaniyah, 12233, Riyadh, Saudi Arabia; Department of Nursing, Ministry of Health (MOH), King Saud Medical City, Ulaishah, Al Imam Abdul Aziz Ibn Muhammad Ibn Saud, 12746, Riyadh, Saudi Arabia; Department of Orthopedic Surgery, Ministry of Health (MOH), King Saud Medical City, Ulaishah, Al Imam Abdul Aziz Ibn Muhammad Ibn Saud, 12746, Riyadh, Saudi Arabia; Department of Orthopedic Surgery, Ministry of Health (MOH), King Saud Medical City, Ulaishah, Al Imam Abdul Aziz Ibn Muhammad Ibn Saud, 12746, Riyadh, Saudi Arabia

**Keywords:** Pipkin I, femoral head fracture dislocation, hip dislocation, skeletal traction

## Abstract

Pipkin I fracture dislocation is a rare injury. They commonly present following high mechanism trauma with possible devastating complications regardless of the treatment option. Treatment consensus depends on fracture reduction, size, site, and displacement. Surgical management is reserved for large fragments. We present a 42-year-old female presented with multiple chest injuries and left sided Pipkin I fracture dislocation Two days following the trauma. The fracture was found to be vertical in nature and was managed by closed reduction and a period of bed rest and skeletal traction. She was followed over a 3-year period and showed satisfactory results. We believe that the vertical nature of the fracture and maintenance of the anatomic reduction led to her successful outcome.

## Introduction

Femoral head fractures are rare entities with a reported incidence of 5%–15% in patients who sustain posterior hip dislocation [1, 2]. It is common for these fractures to be missed in emergency settings, which is known to yield poor outcomes [3]. They affect males predominantly and are associated with high mechanism trauma [4]. Femoral head fractures can result in multiple documented complications such as post-traumatic arthrosis, heterotrophic ossification, and avascular necrosis of the femoral head [5]. After obtaining the appropriate informed consent, we report our management outcome of an extremely rare presenting Pipkin Type I variant where there was a large fracture fragment comprising around 40% of the femoral head with an associated hip dislocation. The patient was treated conservatively and showed a satisfactory outcome over 3-year follow-up.

## Case report

**Figure 1:**
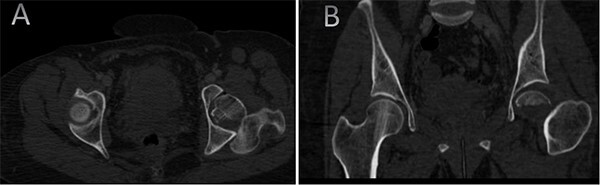
CT scan of the chest, abdomen, and pelvis showing axial (A) and coronal (B) cuts of a left sided Pipkin I fracture comprising large surface of the femoral head with posterior hip dislocation.

**Figure 2:**
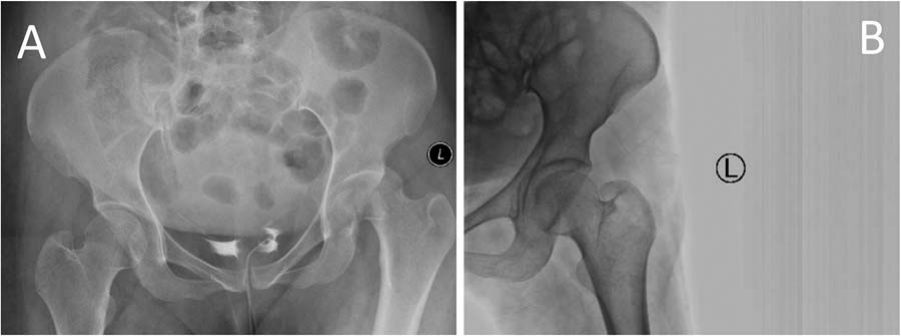
Prereduction anteroposterior (AP) radiographs of the pelvis showing a left side fracture dislocation of the left proximal femur (A) and postreduction radiographs of the left hip (B) showing a concentric hip joint.

**Figure 3:**
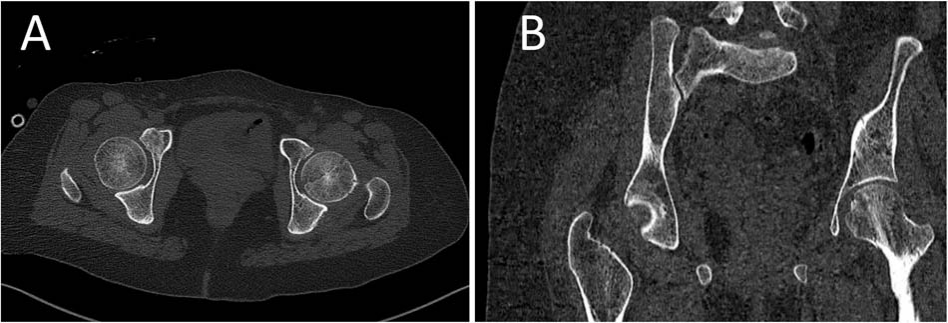
CT scan of the pelvis showing axial (A) and coronal (B) cuts at 6-week post-injury showing signs of healing of the left fractured femoral head with a concentric hip joint.

**Figure 4:**
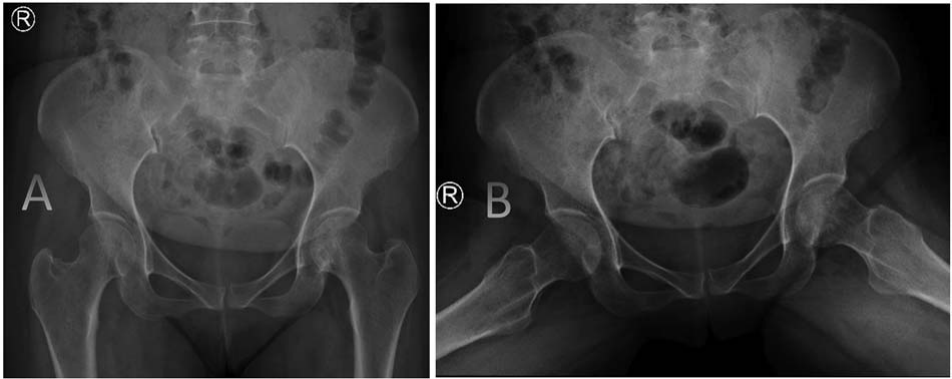
AP radiographs (A) and frog leg lateral (B) radiographs at 3-year follow-up showing a concentrically reduced hip.

A medically free 42-year female presented to our institute as a life-saving case from a different hospital as she was a victim of unrestrained road traffic accident 2 days prior to presentation. Upon assessment in the emergency department (ER), she was conscious and oriented, and was found to have bilateral lung contusion, and multiple fractures of ribs. She also had a left sided vertical femoral head fracture dislocation comprising around 40% of the femoral head (Fig. 1). Closed reduction under conscious sedation was done in the ER, which was successful based on post-reduction imaging studies (Fig. 2). Her chest injuries were treated with chest tube and observation for 10 days. During that time, discussion was made with the patient regarding her situation and conservative management was chosen by way of bed rest and continuation of skeletal traction for 4 weeks with serial radiographs in the hospital on a weekly basis after clearance of her chest injuries. Skeletal traction was discontinued and she was advised to continue bed to wheelchair mobilization for an additional 2 weeks. After 6-weeks from the injury, a Computed Tomography (CT) was done to the patient and revealed signs of fracture healing (Fig. 3). At 3-years after the injury, she was found to have full painless range of motion of the affected hip and has resumed her activity of daily living without any complaints and the images showed a symmetrical joint space of the hips (Fig. 4).

## Discussion

Treatment consensus regarding the management of Pipkin Type I fractures is still yet to be cleared. It was reported that the outcomes are worse following conservative management of Pipkin I fracture with associated hip dislocation compared with surgical excision of the fragment. A report suggests that outcomes are improved with reduction of hip dislocation done within 6 hours from the time of injury [6]. Another report showed that early reduction of a femoral head fracture dislocation resulted in better outcomes [7]. It is expected that delay in reduction of joints will be more challenging and complicated because of contracture and soft tissue oedema. Reduction of the femoral head fracture dislocation can lead to an iatrogenic femoral neck fracture, further displacement, and worsening or comminution of the fracture [2]. Fortunately, concentric reduction was achieved in our patient. Our case was lately presented as a result of multiple transfers until reaching our institute.

A similar case to ours in terms of fracture size, vertical pattern, and associated hip dislocation was reported by Sallameh *et al*. However, a surgical treatment was done to their patient urgently but without a documented long-term follow-up [8]. Availability of adequate service for treating such rare complex injuries is crucial as we believe the delay in transfer of our patient could be result COVID-19 pandemic and focus on her multiple chest injuries rather than her hip. Baidoo *et al*. [9] also reported a similar case to ours in terms of Pipkin I and an associated hip dislocation with a large fragment; however, their fracture pattern was oblique in nature and was not anatomically reduced after closed reduction which led them to do open reduction and internal fixation.

Yoon *et al*. proposed that small or comminuted Pipkin I require surgical excision, whereas large fragments typically require anatomical fragment reduction and fixation [10]. What made us consider offering conservative treatment is that the fragment was anatomically reduced in postreduction computed tomography (CT) scan. Another reason that could have helped at maintaining the reduction is the vertical nature of the fracture, whereas if it was oblique or horizontal, shear forces produced by hip abductors would have displaced the fracture. Moreover, in the prereduction trauma CT, the fragment was found to be positioned well in the corresponding acetabulum likely restrained by iliopsoas, indirect head of the rectus femoris, and the capsule [11]. The patient was followed on a weekly basis with radiographs while on skeletal traction.

A meta-analysis was conducted comparing long-term outcomes of 97 patients with Pipkin I fractures treated using different methods such as fragment excision, conservative management, and open reduction internal fixation. It was found that of the patients managed with closed reduction alone, 67% developed arthritis, 7% developed avascular necrosis, and 2% developed heterotopic ossification. When comparing functional outcomes of conservative management to surgical treatment using the Thomas Epstein score and the Merle d’ Aubigne and Postal score, they reported a poor to worse score of 16% and 36%, respectively [5]. Shakya *et al*. [4], however, found a satisfactory outcome with conservative management of Pipkin Type I fractures with a 28% complication rate compared with 72% of the surgically treated cases. We believe that outcomes in the literature are conflicting one another because femoral head fractures are rare injuries and are not simple fractures that could present identically in every patient as some might present with an associated chondral or labral pathologies and are not commonly investigated.

## Conclusion

Pipkin I injuries are challenging yet rare clinical entities with seemingly each fracture presenting differently. Our decision in offering conservative treatment was based on the anatomic reduction achieved after the trial done in ER along with the vertical nature of the fracture. The fracture was healed on follow-up and the patient showed a satisfactory functional result at 3-year follow-up.

## Consent

Written informed consent was obtained from the patient for publication of this case report and accompanying images. A copy of the written consent is available for review by the editor-in-chief of this journal on request.

## Conflict of interest statement

Both surgical and non-surgical treatment options were discussed with the patient along with complications that could arise in the future necessitating surgical treatment.

## Funding

The study is not funded.

## Data availability

Any required links or identifiers for the data are present in the manuscript as described without any edits that could potentially affect the quality of the scientific message provided.
